# Reducing Fall Risk with Combined Motor and Cognitive Training in Elderly Fallers

**DOI:** 10.3390/brainsci7020019

**Published:** 2017-02-10

**Authors:** Francesco Barban, Roberta Annicchiarico, Matteo Melideo, Alessia Federici, Maria Giovanna Lombardi, Simone Giuli, Claudia Ricci, Fulvia Adriano, Ivo Griffini, Manuel Silvestri, Massimo Chiusso, Sergio Neglia, Sergio Ariño-Blasco, Raquel Cuevas Perez, Yannis Dionyssiotis, Georgios Koumanakos, Milo Kovačeić, Nuria Montero-Fernández, Oscar Pino, Niels Boye, Ulises Cortés, Cristian Barrué, Atia Cortés, Peter Levene, Stelios Pantelopoulos, Roberto Rosso, José Antonio Serra-Rexach, Angelo Maria Sabatini, Carlo Caltagirone

**Affiliations:** 1 Clinical and Behavioral Neurology Laboratory, IRCCS Fondazione Santa Lucia, Rome 00179, Italy; r.annicchiarico@hsantalucia.it (R.A.); a.federici@hsantalucia.it (A.F.); mg.lombardi@hsantalucia.it (M.G.L.); s.giuli@hsantalucia.it (S.G.); c.ricci@hsantalucia.it (C.R.); f.adriano@hsantalucia.it (F.A.); i.griffini@hsantalucia.it (I.G.); silvestri-manuel@hotmail.it (M.S.); massimochiusso@gmail.com (M.C.); c.caltagirone@hsantalucia.it (C.C.); 2 Berlin School of Mind and Brain, Humboldt-Universität zu Berlin, Berlin 10117, Germany; 3 Engineering Ingegneria Informatica SpA, Rome 00185, Italy; matteo.melideo@eng.it (M.M.); sergio.neglia@eng.it (S.N.); 4 Hospital General de Granollers, Barcelona 8400, Spain; sarinoblasco@gmail.com (S.A.-B.); rcuevas@fhag.es (R.C.P.); 5 Social Policy Center, Municipality of Kifissia, Athens-Kifissia 14562, Greece; yannis_dionyssiotis@hotmail.com; 6 Frontida Zois Home Care Agency, Patras 25002, Greece; info@frontidazois.gr; 7 Municipality of Stari Grad, Belgrade 11000, Serbia; milo.kovacevic@starigrad.org.rs; 8 Geriatric Department, Hospital General Universitario Gregorio Marañón, Instituto de Investigación Sanitaria Gregorio Marañón, Madrid 28007, Spain; nmonterof@yahoo.es (N.M.-F.); joseantonio.serra@salud.madrid.org (J.A.S.-R.); 9 Benito Menni CASM, Sant Boi de Llobregat-Barcelona 08830, Spain; opino1@gmail.com; 10 Klinisk Informatik, Aarhus 8000, Denmark; niels.boye@kliniskinformatik.dk; 11 Knowledge Engineering & Machine Learning Group Computer Software Department, Universitat Politècnica de Catalunya-BarcelonaTech, Barcelona 08034, Spain; ia@lsi.upc.edu (U.C.); cbarrue@lsi.upc.edu (C.B.); atia.cm@gmail.com (A.C.); 12 Docobo Ltd., Bookham, Leatherhead KT23 4AA, UK; Peter.Levene@docobo.co.uk; 13 Singular Logic, Athens 145 64, Greece; spantelopoulos@singularlogic.eu; 14 Elettronica Bio Medicale S.r.l., Foligno 06034, Italy; rosso@tesan.it; 15 Facultad de Medicina, Universidad Complutense, CIBERFES, Madrid 28040, Spain; 16 The BioRobotics Institute, Scuola Superiore Sant’Anna, Pisa 56127, Italy; angelo.sabatini@sssup.it; 17 Systems Medicine Department, University of Rome “Tor Vergata”, Rome 00173, Italy

**Keywords:** fall risk, fear of falling, elderly, motor training, cognitive training, executive functions

## Abstract

Background. Falling is a major clinical problem in elderly people, demanding effective solutions. At present, the only effective intervention is motor training of balance and strength. Executive function-based training (EFt) might be effective at preventing falls according to evidence showing a relationship between executive functions and gait abnormalities. The aim was to assess the effectiveness of a motor and a cognitive treatment developed within the EU co-funded project I-DONT-FALL. Methods. In a sample of 481 elderly people at risk of falls recruited in this multicenter randomised controlled trial, the effectiveness of a motor treatment (pure motor or mixed with EFt) of 24 one-hour sessions delivered through an *i*-Walker with a non-motor treatment (pure EFt or control condition) was evaluated. Similarly, a 24 one-hour session cognitive treatment (pure EFt or mixed with motor training), delivered through a touch-screen computer was compared with a non-cognitive treatment (pure motor or control condition). Results. Motor treatment, particularly when mixed with EFt, reduced significantly fear of falling (F(1,478) = 6.786, *p* = 0.009) although to a limited extent (ES −0.25) restricted to the period after intervention. Conclusions. This study suggests the effectiveness of motor treatment empowered by EFt in reducing fear of falling.

## 1. Introduction

Falling is a major clinical problem in elderly people aged 65 and over, affecting 30%–40% of those living in the community and 50% living in nursing homes. Falls may lead to negative consequences such as immobilization and injuries and these consequences reduce mobility, independence, quality of life and life span [1,2]. They also increase the fear of falling, which is related to the risk of falls [3]. Fear of falling is experienced by elderly people after a fall [4] but also by those who have never fallen [5] and this might explain the observed higher percentage of older adults reporting fear of falling than those reporting falls in the previous 3 months [6]. However, fear of falling is related to the production of an inappropriately cautious gait [7] and this might in turn cause falls that result in spiraling risk of falls, fear of falling, and functional decline [3].

During the last twenty years studies on falls have substantially increased. However, the pathophysiology is still not clear and this might be due to the multifactorial etiology of falls [7]. A fall is ‘an unexpected event in which the participants come to rest on the ground, floor, or lower level’ [8]; cause is only clear in 15% of cases (e.g., secondary to syncope, related to a neurological disease, vestibular deficit, muscular weaknesses, an impairment of the afferents systems such as vision or hearing loss, etc.) [9]. All the other conditions are identified as ‘idiopathic fallers’, i.e., subjects who fall without any overt cause [10]. History of previous falls, abnormalities of gait or balance [11] and a reactive stepping behavior in response to forward loss of balance [12] are risk factors for falls. However, other factors such as cognitive and behavioral impairments might be other indicators of risk of falls [7,13]. 

The impairment of executive functions and attention impairs postural control. Addressing the latter can be a strategy, per se, to prevent falls because it might be sufficiently flexibly to adapt to the changing environment [7]. For this purpose, dual task protocols, in which the subjects are asked to simultaneously perform a motor and a cognitive task, proved the reciprocal influence between motion and cognition [14] and its failure is a strong predictor of falls [15]. In fact, postural sway increases when the subject executes a cognitive task [16,17] demonstrating that attention is required to control posture. The relationship between motor and cognitive abilities plays an important role in falls of elderly people since age decreases sensory information and increases the demand for greater attention in postural control [18]. In fact, the reduction of attention and executive functions seems to be a primary cause in idiopathic fallers [19] and appears to be an important risk factor for falls [20] in subjects with a non-amnestic mild cognitive impairment (MCI), based primarily on executive functions. 

Concerning prevention strategies for the risk of falls, at present motor training of balance and strength appears to be the only intervention program that reduces both the number of fallers and the number of falls in community dwellers [21]. In particular, providing intensive balance exercise seems to be effective in reducing falls [22]. Physical training also has an indirect effect on falls prevention through a positive effect on cognitive abilities [23,24,25,26]. Other beneficial approaches are: the home hazards modification, especially in high-risk groups; drugs adjustment and some surgical interventions such as cataract and pacemaker implantation [21]. Recent evidence also supports the beneficial effect of cognitive training on falls reduction [27]. In particular, training to enhance attention and executive function produce improvements of gait [28] and in the elderly the combination of motor and cognitive training using Information and Communication Technologies (ICT) generates improvement of physical functioning [28,29]. All these results encourage future research since the heterogeneity of the previous studies does not allow being conclusive in this regard. 

The present randomised controlled trial (RCT) is part of the multicenter and international I-DONT-FALL (IDF) project co-funded by the European Union, offering an integrated system for fall risk prevention and detection. Participants were randomised into a single cognitive or motor training, a combined training and an active control condition, all of them lasting for 24 one-hour sessions (twice-a-week) and were tested before and after the training period and then after a follow-up period using standardised scales assessing mobility, cognitive abilities, behavior and functional aptitudes. 

The primary aim of this study was to test the impact of these different training types on fall risk in elderly people at risk of fall. Consequently, this required testing the hypothesis that motor and mixed training would: (1) reduce the fear of falling; (2) increase balance and gait abilities. The secondary outcome was to examine the impact of these different training approaches on the cognitive, behavioral and functional domains. This meant that a test of the hypothesis that cognitive and mixed training would: (1) increase cognitive abilities; (2) improve behavior (mood and anxiety); (3) increase functional abilities.

## 2. Experimental Section

### 2.1. Methods

#### 2.1.1. Study Design and Randomisation

**Figure 1:**
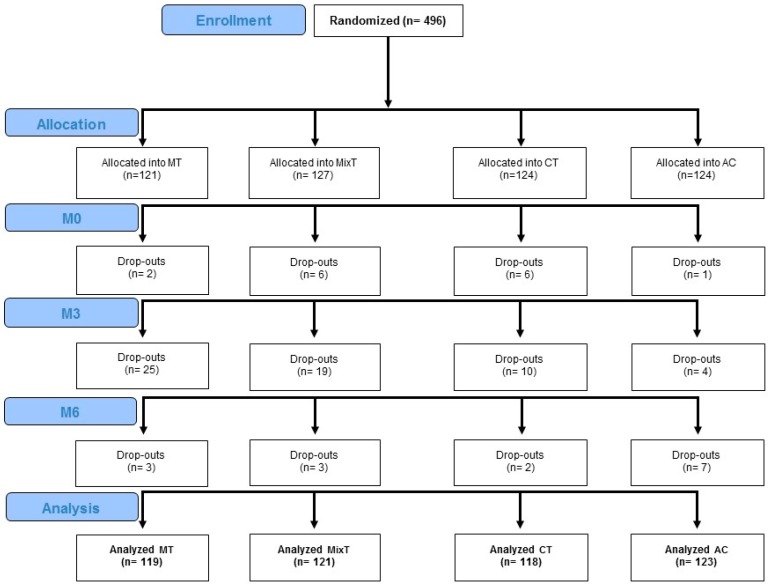
Flow chart of participants in the study showing the allocated and analyzed participants and drop-outs in the four arms of the study and the three multidimensional assessments before: the onset of the treatment, at the end of the treatment after 3 months (M3) and after other 3 months of follow-up (M6). Abbreviations: MT: motor treatment; MixT: mixed treatment; CT: cognitive treatment; AC: active control.

This was a multicentre, stratified, double-blind, controlled, parallel-group study conducted in Italy, Greece, Spain and Serbia. For allocation of participants a concealed [30] computer-generated blocked (blocks size: 6) randomisation [31] was used. It was stratified by pilot sites in two steps: firstly, subjects were randomised between the presence/absence of motor training and then between the presence/absence of cognitive training. This resulted in 4 different arms: only motor training (MT), only cognitive training (CT), mixed motor and cognitive (MixT), active control (AC) and those not receiving either cognitive or motor training. A central randomisation service of an independent pilot site sent the allocation of each participant via Internet to the investigators responsible for recruitment. After being randomised between the different arms of the study, each participant underwent a complete multidimensional evaluation (mobility, cognitive, behavioural and functional) at baseline at month zero (M0), after 3 months during which subjects underwent 24 one-hour sessions of treatment twice-a-week (M3) and after a follow-up period of 3 months (M6) (see Figure 1). Expert clinicians for each pilot site, different from those responsible for enrolment and blind about the allocation of the participant, conducted the assessments.

#### 2.1.2. Subjects

The study comprised 496 enrolled subjects of which 481 were included in the final analyses. All participants were enrolled in seven centres that included hospitals or local municipality centres in Italy, Greece, Spain and Serbia. They all underwent a clinical screening that included the collection of medical history, history of previous falls and the administration of the Tinetti Performance Oriented Mobility Assessment (POMA) [32] and the Mini Mental State Examination (MMSE) [33]. Eligible participants were elderly (aged ≥ 65 years) with a formal education of at least 5 years who met the inclusion eligibility criteria for risk of fall according to previous studies [34,35,36] (total POMA score ≤ 20 and/or at least one fall in the previous year). Exclusion criteria were: the presence of major cognitive (MMSE ≥ 20) disturbances, history of behavioral, psychiatric and/or systemic disturbances and/or receiving any rehabilitative treatment. From the initial sample, 73 participants (14.7%) dropped-out before the end of the training period and 15 subjects did not complete the follow-up evaluation (Figure 1). In the final analyses 481 participants were included (i.e., all the enrolled patients with the exception of the 15 subjects that did not executed the baseline (M0) assessment). All participants provided their written informed consent approved by the local Ethics Committee of each pilot site. 

#### 2.1.3. Trainings and Active Control Condition

All the treatment forms were administered through 24 one-hour sessions twice-a-week. They occurred in an inpatient setting or at a participant’s home. The intervention developed in this study comprised both motor and cognitive exercises and these were administered accordingly using the abovementioned randomization. Those participants randomised into MT underwent pure motor training, consisting in a set of warm-up procedures (i.e., stretching and squat) followed by exercises dedicated for half of the time of each session to balance and half to gait. These were administered through an *i*-Walker [37], an assistive technology device developed with the aim to support users with mobility disturbances by compensating unbalanced muscle force and lack of muscle force on climbs and descents. Balance training consisted of exercises lifting up heels or tiptoes, lateral/forward shifting, holding and flexion/extension exercises. Gait exercises involved moving the *i*-walker forward and backward with several variants. All exercises were augmented in difficulty by increasing speed, repetition, changing holding position or with one handed use of *i*-walker handles (see Appendix (Table A1 and Table A2) for a description of each balance and gait exercise). Participants randomised into CT underwent a set of exercises mainly focused on executive functions and attention (2/3 of the time of each training session). These were provided by trained cognitive therapists in an individual or group setting (up to three participants per session) administered through a computerized touch-screen platform (either in a table or in an all-in-one desktop computer) developed within a project called SOCIABLE [38] co-funded by the European Union [39]. Each exercise provided three increasing levels of difficulty adjusted by the therapists accordingly to the subject’s capability (i.e., each exercise was set at a higher level of difficulty after two consecutive correct sessions). In particular, executive function exercises consisted of working memory, planning, and abstraction tasks (e.g., ordering at restaurant following some rules, solving tasks on similarities, differences and analogies, sorting pictures guessing a covered criterion), whereas attention exercises consisted of selective and sustained attention tasks (e.g., paying attention to a target item among distractors). Exercises of other main cognitive functions (i.e., declarative memory, orientation, constructional praxis, language and abstract reasoning) were executed during the remaining ⅓ of the time of each training session. MixT comprised both of the abovementioned treatments resulting in 30 min of CT and 30 min of MT per session. Finally, AC consisted of entering data into the same platform used during the CT. Data consisted of words, names, addresses, telephone numbers, dates of birth, personal codes, names of towns, bank codes, and non-words. This activity involved only automatic cognitive processes (i.e., reading and writing), and not higher order cognitive functions [40].

### 2.2. Outcomes

The primary outcome of this study was the assessment of the impact of different training on risk of falls measured with standardized scales assessing:

Mobility: Evaluation of balance and gait with Tinetti Performance Oriented Mobility Assessment (POMA) [32] for balance (POMA-B) and gait (POMA-G) and the fear of falling through Falls Efficacy Scale—International (FES-I) [41].

The secondary outcome of this study was the assessment of the impact of different trainings on cognitive, behavioral and functional aptitudes. These were measured with standardized tests and scales assessing:

Cognition: Evaluation of executive functions and attention with Trail Making Test (TMT) [42], phonological fluency test (PF) [43] and of verbal and visuo-spatial memory with Rey Auditory Verbal Learning Test (RAVLT) [44] and Rey-Osterrieth Complex Figure test (ROCF) [45].

Behavior: Evaluation of mood with Geriatric Depression Scale (GDS) [46] and anxiety with State-Trait Anxiety Inventory—Y (STAI-Y), both State and Trait scale [47]. 

Daily functioning: Evaluation of daily functioning with Instrumental Activities of Daily Living scale (IADL) [48] and Barthel Index (BI) [49].

### 2.3. Sample Size

The sample size was based on a previous intervention study [50] assessing the effect of balance training on fear of falling with FES-I. It was estimated that the minimum total sample size would be 447 (based on a = 0.05, power = 0.80, four groups) [51]. This compares favorably with the total sample size of 481.

### 2.4. Statistical Analysis

In the final analyses, only the participants that did not complete the baseline assessment were excluded. The multiple imputation technique for analyzing incomplete data sets to generate the dataset to perform the analyses was adopted. This was the average (pooled dataset) of five imputed datasets generated with SPSS (SPSS Inc., Chicago, IL, USA).

To assess possible differences at baseline (M0) between the samples receiving different treatments, for each outcome and demographic variable two one-way ANOVAs were performed comparing motor vs. non-motor condition and cognitive vs. non-cognitive condition (Table 1). 

To test the experimental hypotheses, for each outcome measure assumed as a dependent variable, a mixed analysis of variance (ANOVA) was performed to test the kind of treatment (motor vs. non-motor and cognitive vs. non-cognitive) and time (M0 vs. M3, i.e., pre vs. post treatment) as main factors and their interaction. Motor condition comprised both MT and MixT, whereas non-motor comprised CT and AC. Cognitive condition comprised both CT and MixT, whereas non-cognitive comprised MT and AC. For each outcome measure, possible follow-up effects were also evaluated by assessing the interaction between the kind of treatment and all of the time points (M0, M3 and M6). To correct for multiple comparisons, the Bonferroni method was applied assuming that within each domain, variables would be more or less dependent on each other and the significant threshold was fixed at *p* < 0.0125. Post-hoc comparisons were executed for significant interactions with two paired *t*-tests comparing time (M0 vs. M3 and M3 vs. M6) separately for each different treatment (MT, CT, MixT, AC). The Bonferroni method was used to correct the post-hoc comparisons and the significant threshold of *p* < 0.0125 was fixed. Cohen d’ effect sizes were calculated dividing the post-pre training difference by the pooled standard deviation. According to Cohen [52] effect sizes around 0.20 are considered small, around 0.50 medium and around 0.80 large.

## 3. Results

### 3.1. Baseline

Table 1 reports descriptive statistics of demographic variables and the study outcomes at baseline. Only a significant difference in the distribution between females and males emerged comparing motor vs. non-motor training and this factor entered the ANOVAs analyses as covariate of no interest for the motor vs. non-motor training comparison.

### 3.2. Treatments effects

Table 2 reports all effects of the motor and cognitive trainings after the study period and at follow-up. The significant ones are described here.

### 3.3. Mobility

**Figure 2:**
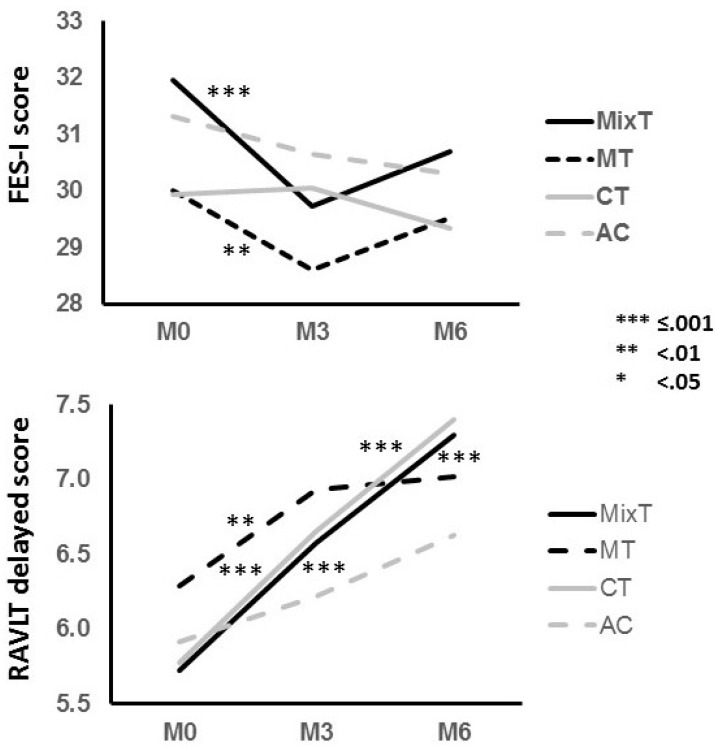
The figure represents the average performance at the Falls Efficacy Scale—International (FES-I) and at the delayed recall of the Rey Auditory Verbal Learning test (RAVLT) at baseline (M0), after the 3 months at the end of the treatment (M3), and after the next 3 months of follow-up (M6). The different lines indicate the four different kinds of treatment: mixed (MixT), motor (MT), cognitive (CT) and the active control (AC). Stars indicate the p-level of significant results at the analysis of variance.

The motor treatment significantly reduced the fear of falling, measured with the FES-I scale, as showed by the significant interaction between time (M0 vs. M3) and treatment (motor vs. non-motor) that was significant at follow-up (see Figure 2, Table 2 and Table 3). Post-hoc investigations revealed that the MixT showed the largest effect followed by the MT, which did not last after the end of the treatment. CT and AC showed no significant effects. All kinds of intervention increased balance and gait, without any significant interactions among them but showed a significant main effect of time.

### 3.4. Cognition

The cognitive treatment significantly increased verbal memory (a trend was present for visuo-spatial memory) as showed by a significant interaction at follow-up (see Figure 2, Table 2 and Table 3) between time (M0, M3 and M6) and treatment (cognitive vs. non-cognitive). Interestingly, post-hoc investigations revealed that the MixT showed a slightly higher effect size than the CT and that also the MT showed a significant positive effect. All kinds of intervention improved similarly executive functions without any significant interactions among them and showing a significant main effect of time. 

### 3.5. Behavior and Functional Abilities

We did not obtain any significant interactions for GDS but only a main effect of time showing a similar reduction of depressive symptoms among the different treatments. Anxiety showed a trend toward a significant interaction in the trait STAI-Y between time (M0 vs. M3) and treatment (motor vs. non-motor) showing a greater reduction of trait anxiety after the motor vs. non-motor training. 

## 4. Discussion 

The effectiveness of motor treatments on fall prevention is widely confirmed by several studies [22,53]. Particularly, motor treatments training balance and strength [22,53,54] are promising in preventing falls, as an impairment of these domains is recognized to play a pivotal role in increasing the risk of falls [55]. However, a recent systematic review by Kearney and colleagues [56] highlights the link between executive dysfunctions and gait abnormalities in older adults although its nature is still unclear. For this reason, a training focused on executive functions might exert some positive effects on mobility and vice versa. A particular important intervention outcome is the umbrella term for a complex pattern of problems called ‘fear of falling’ that includes fear, anxiety, loss of confidence and self-efficacy, avoidance behavior, social isolation that all contribute to increase frailty. It must be noted that fear of falling is found in elderly who fall but also in those who have never fallen [4,5,54].

The present study investigated the separate and conjoint effect of a balance/gait motor training and an executive function cognitive training on mobility, cognitive, behavior and functional outcomes in a large sample of elderly at risk of falls. Results confirm two findings already present in literature [57,58]. The motor training significantly reduced the fear of falling with a tendency to reduce the correlated anxiety. On the other hand, the cognitive training significantly improved cognitive abilities, in particular episodic memory. To clarify, two memory tests, based on the recall process, were used that also require executive functions to be performed [59]. The most suggestive result comes from the comparison between the single and the conjoint training showing that, for mobility and slightly also for cognitive variables, the mixed training showed stronger effects than the single ones. Although recent studies showed that cognitive approaches can also reduce the fear of falling [60], the results suggest that training patients conjointly on mobility and cognitive abilities produces better results than substantial training on exercises only involving one aspect. This might be the result of a more stimulating training in term of motivation or that the conjoint motor and cognitive stimulation might involve different mechanisms in the brain that produce better results. 

However, our results are far to be conclusive. In fact, accordingly to recent Cochrane reviews assessing the effects of exercise interventions for reducing fear of falling in elderly people [61,62], our intervention reduced fear of falling to a limited extent (ES −0.25) and the effect was restricted to the period immediately after the end of the intervention. Nevertheless, although our effect represents only a small difference [52], there is still not a clear consensus on the definition of minimal clinically important difference for fear of falling measures. Taking into consideration this specific research context, all previous studies included in the Cochrane reviews [61,62] that implied as outcome the FES used the original 10-item [63,64,65,66]. They reported heterogeneous ESs (small, medium, very large) as well as different FES baseline scores and, by consequence, populations with different fear of falling levels. Interestingly, a recent study [67] implied the FES-I to assess the effectiveness of a two-year exercise programme of progressive balance retraining in elderly women at risk of falls. Authors reported that fear of falling increased during the study but less in the intervention group (about 2 points during the first year) than in the control one (about 7 points during the first year). Although our effects were small in magnitude, it is so far not clear the clinical value to take into consideration for their correct interpretation [68] and this deserve further investigations. 

The present study did not show any strong behavioral and functional effects after any treatments. This might be due to the low impact of these deficits in the population that was recruited for this study and a full significant effect might emerge in more severely impaired populations. 

Finally, a quite unspecific improvement, in most outcomes as shown by the significant effect of time in the employed measures, was observed. Although this might be a confound for the statistical analyses, it also constitutes a positive result suggesting that the simple activity of taking care of elderly people at risk of falls might exert some positive effects on their mobility and cognitive abilities. However, this consideration deserves some further studies. 

## 5. Conclusions

Consistently with data previously reported in literature, this study shows the effectiveness, although to a limited extent to the period immediately after the end of the intervention, on fear of falling of motor training focused on gait and balance exercises. It also shows the effectiveness of cognitive training focused mainly on executive function exercises on cognitive outcomes and it suggests that a combination of motor and cognitive treatment tends to maximize all these effects, thus confirming the strength of the link between the domains of gait and balance performance and executive functions. However, the underlying mechanism of this effect is still unclear and the lack of clear clinical values in this research context makes the interpretation of the magnitude of the effects of the treatment difficult. For these reasons, further studies are needed to clarify these issues. 

## Figures and Tables

**Table 1:** Demographics and study outcomes at baseline.

		Motor		Non-Motor					
		Non-Cognitive	Cognitive	Cognitive	Non-Cognitive				
Kind of Treatment		MT	MixT	CT	AC	M/noM		C/noC	
		*N* = 119	*N* = 121	*N* = 118	*N* = 123	*χ^2^* F	*p*	*χ^2^* F	*p*
Sex f(m)		82(37)	87(34)	65(53)	80(43)	5.575	0.018	0.593	0.441
Domain	Variable	m(sd)	m(sd)	m(sd)	m(sd)				
Demographic	Age (years)	75.5(8.5)	74.5(7.9)	74.1(7.2)	76(8.8)	0.002	0.969	0.428	0.513
	Education (years)	9.7(4.3)	10.2(4.8)	9.9(4.2)	10(4.2)	0.000	0.991	0.653	0.419
Mobility	FES-I	30(10.2)	32(9.3)	29.9(9.7)	31.3(11)	0.145	0.703	0.102	0.749
	POMA B	11.9(3.4)	11.1(3.5)	11.5(3.3)	11.3(3.5)	0.116	0.734	1.076	0.300
	POMA G	8.7(2.6)	8.1(2.8)	8.2(2.6)	8.2(2.9)	0.390	0.533	1.479	0.224
Cognitive	TMT B-A	117.7(72.2)	135.4(76.1)	119.5(68.3)	117(65.5)	1.699	0.193	2.502	0.114
	PF	25.7(12.4)	24.8(12.3)	24.4(12.6)	25.2(10.8)	0.185	0.667	0.560	0.445
	RAVLT d	6.3(3.8)	5.7(3.5)	5.8(3.7)	5.9(3.5)	0.206	0.650	1.106	0.293
	ROCF d	10.1(7.4)	8.2(6.7)	9.9(7.2)	7.9(6.5)	0.195	0.659	0.002	0.963
Behavioral	GDS	5.2(3)	5.6(3.4)	5(3)	5.8(3.2)	0.005	0.946	0.344	0.558
	STAI-Y s	36.4(10.1)	37.1(10.7)	36.4(10.2)	36.4(10.1)	0.146	0.702	0.176	0.675
	STAI-Y t	39.6(9.9)	39.7(9.9)	38.8(9.9)	39(9.6)	0.678	0.411	0.002	0.964
Functional	BI	86(19.9)	84.6(20.7)	86.6(19.7)	86.1(18)	0.328	0.567	0.061	0.804
	IADL	6.3(2.3)	6.2(2.4)	6.2(2.2)	5.9(2.5)	0.755	0.385	0.197	0.657

Abbreviations: MT: motor training; MixT: mixed training; CT: cognitive training; AC: active control; M: motor; noM: non-motor; C: cognitive; noC: non-cognitive; f(m): female(male); m(sd): mean (standard deviation); POMA B/G: Performance Oriented Mobility Assessment of balance/gait; FES-I: Falls Efficacy Scale-International; RAVLT d: Rey Auditory Verbal Learning Test, delayed recall; ROCF d: Rey-Osterrieth Complex Figure, delayed recall; TMT B-A: Trail Making Test B-A; PF: Phonological Fluency test; STAI-Y s/t: State-Trait Anxiety Inventory; GDS: Geriatric Depression Scale; BI: Barthel Index; IADL: Instrumental Activities of Daily Living. *χ*^2^: Pearson chi square; F: F-ratio of the analysis of variance; *p*: *p*-value.

**Table 2:** Results.

		Motor/Non-Motor *								Cognitive/Non-Cognitive							
		Time		Group		Int		FU-Int		Time		Group		Int		FU-Int	
		F	*p*	F	*p*	F	*p*	F	*p*	F	*p*	F	*p*	F	*p*	F	*p*
*Mobility*	FES-I	2.553	0.111	1.030	0.311	6.786	0.009	4.900	0.009	13.564	<0.001	0.108	0.743	0.004	0.952	0.059	0.934
	POMA B	37.422	<0.001	1.166	0.281	3.442	0.064	2.016	0.146	61.258	<0.001	1.219	0.270	0.000	0.999	0.155	0.797
	POMA G	5.626	0.018	0.599	0.439	0.005	0.943	0.318	0.707	24.402	<0.001	1.016	0.314	0.901	0.343	0.590	0.539
*Cognition*	TMT B-A	3.564	0.060	1.149	0.284	0.276	0.599	0.225	0.788	15.247	<0.001	1.833	0.176	1.216	0.271	1.292	0.275
	PF	7.450	0.007	0.302	0.583	0.185	0.667	1.403	0.247	7.634	0.006	0.367	0.545	0.445	0.505	0.277	0.744
	RAVLT d	20.425	<0.001	0.379	0.538	0.845	0.358	0.517	0.583	53.692	<0.001	0.238	0.626	4.699	0.031	9.040	<0.001
	ROCF d	5.148	0.024	0.813	0.368	0.369	0.544	0.171	0.829	28.979	<0.001	0.374	0.541	5.048	0.025	2.411	0.094
*Behavior*	GDS	8.374	0.004	0.145	0.704	0.176	0.675	0.427	0.632	13.878	<0.001	0.245	0.621	0.170	0.680	0.575	0.546
	STAI-Y s	2.234	0.136	0.077	0.782	0.705	0.402	0.551	0.564	1.561	0.212	0.031	0.861	0.824	0.364	1.241	0.288
	STAI-Y t	0.020	0.888	0.066	0.798	5.649	0.018	2.738	0.069	0.008	0.928	0.000	0.998	.023	0.881	0.506	0.592
*Function*	BI	2.497	0.115	0.011	0.916	1.578	0.210	1.219	0.279	11.288	0.001	0.004	0.948	0.868	0.352	1.483	0.227
	IADL	5.630	0.018	0.656	0.419	0.051	0.821	0.111	0.858	9.713	0.002	0.162	0.687	0.080	0.777	0.518	0.561

Abbreviations: Int: interaction; FU: follow-up; F: F-ratio of the analysis of variance; *p*: *p*-value (significant *p*-values are in bold); FES-I: Falls Efficacy Scale-International; POMA B/G: Performance Oriented Mobility Assessment of balance/gait; TMT B-A: Trail Making Test B-A; PF: Phonological Fluency test; RAVLT d: Rey Auditory Verbal Learning Test, delayed recall; ROCF d: Rey-Osterrieth Complex Figure, delayed recall; GDS: Geriatric Depression Scale; STAI-Y s/t: State-Trait Anxiety Inventory; BI: Barthel Index; IADL: Instrumental Activities of Daily Living. Degrees of freedom of ANOVAs: F(1,478) and for follow-up F(2,956). * All F tests of the motor/non-motor with sex as covariate of no interest.

**Table 3:** Post-hoc results for significant interactions.

Treatment	Test	Pre	Post	FU	Pre-Post		ES	Post-FU		ES
					t	*p*		t	*p*	
MT	**FES-I**	30(10.2)	28.6(9.2)	29.5(9.5)	2.777	**0.006**	−0.14	−1.773	0.079	0.10
MixT		32(9.3)	29.7(8.5)	30.7(8.7)	3.889	**<0.001**	−0.25	−2.181	0.031	0.11
CT		29.9(9.7)	30.1(9.4)	29.3(9.9)	−0.187	0.852	0.01	1.293	0.199	−0.07
AC		31.3(11)	30.6(10.5)	30.3(10.3)	1.245	0.215	−0.06	0.716	0.475	−0.03
MT	**RAVLT d**	6.3(3.8)	6.9(3.5)	7(3.7)	−2.780	**0.006**	0.17	−0.443	0.659	0.03
MixT		5.7(3.5)	6.6(3.3)	7.3(3.2)	−5.986	**<0.001**	0.25	−3.555	**0.001**	0.22
CT		5.8(3.7)	6.7(3.7)	7.4(3.8)	−5.782	**<0.001**	0.24	−3.405	**0.001**	0.20
AC		5.9(3.5)	6.2(3.3)	6.6(3.3)	−1.593	0.114	0.09	−2.076	0.040	0.12

Abbreviations: FU: follow-up; t: Student’s t; *p*: *p*-value (significant *p*-values are in bold); ES: effect size; MT: motor training; CT: cognitive training; MixT: mixed training; AC: active control; FES-I: Falls Efficacy Scale-International; RAVLT d: Rey Auditory Verbal Learning Test, delayed recall.

## Appendix A

**Table A1:** Cognitive exercises of I-DONT-FALL (IDF) cognitive training.

Exercise	Contents	Objective of training
**Memory**		
Remember the picture	The user is shown a picture, then this is immediately replaced with other pictures. The user is asked to find the previous picture among the subsequent ones.	Declarative episodic short-term visuo-spatial memory
Remember the melody	The user is presented with a piano playing some notes with corresponding key lighting up. The user is asked to reproduce the melody.	Declarative episodic short-term auditory memory
Hide and find	The user is shown one of three fully-furnished rooms (living room, bathroom, kitchen). During the encoding phase the user is asked to hide from 5 to 10 items in there (in the room). After 15 minutes, during the delayed recall phase, the user is asked to reposition the items in their initial location.	Declarative episodic long-term visuo-spatial memory
Do you remember your order	The user is shown two menus (his and his friend’s) and is asked to memorize them. Immediately after, the user is asked to recognize the ordered dishes among distracters.	Declarative episodic short-term verbal memory
Remember the design	The user is shown a design drawn on a 9-dot matrix and is asked to encode it. Immediately after the design disappears, the user is asked to reproduce it on the same 9-dot matrix.	Declarative episodic short-term visual memory
Find the pairs	The user is shown a grid of uncovered paired cards. After the cards are covered, the user is asked to find the pairs of identical pictures.	Declarative episodic short-term visual memory
Who belongs where	The user has to choose the correct profession of famous people among two alternatives.	Semantic memory
**Executive functions and attention**		
Similarities	The user is asked to select the correct sentence among 3 describing the similarity between two concepts.	Abstraction
Differences	The user is asked to select the correct sentence among 3 describing the difference between two concepts.	Abstraction
Analogies	The user is given one pair of related concepts and another concept without its pair. The subject is asked to choose the correct concept out of four to form another pair.	Abstraction
Picture sort	The user is presented a series of pictures and is asked to move each one into one of two boxes following a rule. This must be inferred by the feedback given each time a picture is moved into the correct or incorrect box.	Abstraction
Be a piano player	The user is presented with a piano playing some notes. When a note plays the corresponding key lights up and the user is asked to press the key and reproduce the note. Notes follow one another at growing speed.	Attention
Take away menu	The user must order a take away menu following some rules (e.g., maximum price, foods not to be ordered).	Inhibition, planning, problem-solving
Train guidance	The user is presented with a train rail and some parts of this are interrupted or missing. The user is asked to straighten or complete the rail in order to ensure the train passage.	Planning, problem-solving
Lost in the city	The user must pay attention to the direction given by the central person among five giving different directions.	Selective attention—interference inhibition
Guess Who	The user is asked to identify the mystery character by excluding those characters who do not meet the descriptions of the right one.	Visuo-spatial attention
N-back	The user is presented with a sequence of pictures and is asked to indicate when the current picture matches the one from n steps earlier in the sequence.	Working memory
Remember the sequence	The user is shown a sequence of pictures. These are positioned in the array each time in a different sequence. The user is asked to remember the pictures in their right sequence. At the same time, if a picture that follows the specified rule is placed in the sequence, the user must clap his hands.	Working memory
**Constructional praxis**		
Puzzle	The user is asked to combine the pieces of a puzzle to complete the final figure	Constructional praxis
Copy of figures	The user is asked to copy the geometrical figures.	Constructional praxis
**Language**		
Synonymous	The user must connect words of two different lists coupling synonymous words.	Lexicon access
Antonymous	The user must connect words of two different lists coupling antonymous words.	Lexicon access
**Logical reasoning**		
Incomplete grids	The user is asked to complete the grid by inserting the right tile in a multiple choice.	Logical reasoning
Symbol addition	The user is asked to solve arithmetic operations using symbols instead of digits.	Logical reasoning
Domino	The user is asked to pair identical images by placing each tile next to the corresponding one.	Logical reasoning
**Orientation**		
My home	The user must move a person into a house following a precise trail.	Spatial orientation
Travelling in Europe	The user is shown a map of Europe and must select different countries according a specified order during a trip.	Spatial orientation

**Table A2:** Balance and Gait exercises

Warm-up pool	Balance pool	Gait pool
Stretching	Lift up heels	Moving i-walker forward
Squat with spread legs	Lift up tiptoes	Moving i-walker forward oblique
Squat with spread legs in anteroposterior	Lift up heels/tiptoes	Moving i-walker forward flexing torso
	Lateral load shift	Moving i-walker forward oblique flexing torso
	Lateral load shift with contralateral leg flexion	Load shift with arms
	Lateral load shift with contralateral leg flexion	Load shift with arms and kick
	and torso rotation	Move i-walker forward / backward
	Forward load shift	Move i-walker forward / backward in line
	Hip lift up opposite the support leg	Move i-walker forward / backward marching
	Load holding for 10 seconds	Move i-walker on a wide curve
	Load holding with heel lift up	Move i-walker on a curve marching in place
	Leg flexion / alternate leg flexion	
	Leg flexion and extension / alternate leg	
	flexion and extension	
	Leg flexion and extension backwards	
	Foot sliding forth and back	
